# Physical activity determinants, 24-h movement behaviors, and cardiovascular fitness in young adults: an integrated theoretical model perspective

**DOI:** 10.3389/fpsyg.2026.1810709

**Published:** 2026-05-28

**Authors:** Dandong Gu, Zarmina Amin, John Oginni, Jessh Mavoungou, Zan Gao

**Affiliations:** 1 School of Physical Education, Hengyang Normal University, Hengyang, China; 2 Department of Kinesiology, Recreation, and Sport Studies, University of Tennessee-Knoxville, Knoxville, TN, United States

**Keywords:** decisional balance, movement behavior, physical activity levels, process of change, sedentary, self-efficacy, sleep

## Abstract

**Purpose:**

Guided by Social Cognitive Theory and the Transtheoretical Model, this study examined differences in physical activity (PA) determinants, 24-h movement behaviors, and cardiovascular fitness (CRF) across stages of change among Chinese college students. Associations between PA determinants, movement behaviors, and CRF were also examined.

**Methods:**

Participants were 220 Chinese college students (105 males; *M* age = 20.15 ± 1.01 years). Students completed validated questionnaires assessing PA determinants (i.e., PA strategies, decisional balance, self-efficacy, social support, enjoyment, and perceived environment). One week of 24-h movement behaviors (sedentary time, light PA, moderate-to-vigorous PA [MVPA], sleep duration and sleep efficiency) was assessed using ActiGraph Link accelerometers. CRF was assessed using a standardized 3-min step test.

**Results:**

Participants classified in higher stages of PA behavior change reported significantly more favorable PA determinants than those in lower stages, including higher self-efficacy (*p* < 0.01, *ƞ*^2^ = 0.12), decisional balance (*p* < 0.01, *ƞ*^2^ = 0.034), PA strategies (*p* < 0.01, *ƞ*^2^ = 0.05), friend support (*p* < 0.01, *ƞ*^2^ = 0.06), and enjoyment (*p* < 0.01, *ƞ*^2^ = 0.07). No significant stage-group differences were observed for accelerometry-derived measured movement behaviors or CRF. Regression analyses showed that MVPA (*t* = 1.96, *p* = 0.05) and self-efficacy (*t* = 1.84, *p* = 0.06) were the strongest contributors to CRF. Light PA (*t* = 16.73, *p* < 0.01) positively predicted with MVPA, whereas sleep efficiency was negatively predicted with MVPA (*t* = −2.00, *p* < 0.01). Additionally, MVPA and sedentary behavior (*t* = −4.87, *p* < 0.01) were significant negative predictors for sleep efficiency.

**Conclusion:**

College students with high stages of PA behavior change demonstrated more positive psychological determinants of PA, though objective activity levels and fitness did not differ. MVPA and self-efficacy emerged as key influences on CRF, highlighting the importance of motivational and behavioral regulation strategies in college-based PA promotion and support the development of stage-tailored, theory-informed PA interventions for college students.

## 1 Introduction

Despite extensive evidence supporting the health benefits of regular physical activity (PA; Ekelund et al., 2024; Hassan et al., 2024; Liang et al., 2024; Stamatakis et al., 2024; Tarp et al., 2024), a substantial proportion of Chinese youth and adults remain insufficiently active and often disengaged from structured exercise programs (Sun, 2016). Among college populations, declining participation has been partly attributed to low motivation—defined as the internal drive that initiates, sustains, and directs behavior (Pintrich and Schunk, 1996). Motivation influences effort, activity choice, and engagement, underscoring the need to better understand its determinants in this demographic.

Recent Chinese studies highlight motivation as a key predictor of PA and health outcomes. Li et al. (2022) demonstrated that motivation predicts cardiovascular fitness (CRF) both directly and indirectly through PA levels, with health-oriented motives exerting the strongest influence. From a social-cognitive perspective, Khairani and Zhang (2025) found that self-efficacy, outcome expectations, social support, and self-regulation significantly predict PA levels among Chinese college students, with notable gender-related differences. Together, these findings suggest that motivation-related constructs are central to understanding PA engagement in Chinese college populations.

To comprehensively address behavioral change, this study adopts an integrated theoretical framework combining Social Cognitive Theory (SCT) (Bandura, 1986) and the Transtheoretical Model (TTM) (Prochaska and Diclemente, 1982). SCT emphasizes reciprocal determinism, describing the dynamic interaction among personal factors, behavior, and environmental influences. Within this framework, self-efficacy is a critical determinant of PA initiation and maintenance, particularly for overcoming barriers associated with sedentary behavior. Enjoyment, peer support, and access to supportive environments further reinforce sustained engagement in PA, especially within campus settings (Bandura, 1986). Collectively, SCT provides a practical structure for understanding how individual, social, and environmental factors shape PA behavior among college students.

TTM offers a complementary, stage-based approach to understanding PA adoption and maintenance (Prochaska and DiClemente, 1982). TTM categorizes individuals into stages of change—precontemplation, contemplation, preparation, action, maintenance, and termination—based on readiness to modify behavior (Marcus and Forsyth, 2003). Individuals may progress or regress across stages, reflecting the non-linear nature of behavior change. Interventions tailored to stage-specific characteristics have been shown to improve engagement and reduce resistance (Prochaska and DiClemente, 1982; Edberg, 2007). Recent evidence supports the applicability of TTM across diverse populations. A 2025 systematic review reported that stage-matched interventions enhanced PA levels and progression through stages of change among children and adolescents (Xie et al., 2025). Additional studies among Japanese adults (Oba et al., 2024) and Korean adults with physical disabilities (Kang et al., 2022) demonstrated that self-efficacy, decisional balance, and processes of change are stage-specific and strongly associated with PA behavior.

Although SCT and TTM have each been widely applied to understand PA among college students, few studies have integrated these frameworks to examine how central determinants, such as self-efficacy, decisional balance, social support, and processes of change, relate to 24-h movement behaviors and CRF in Chinese college students. Recent evidence suggests that multi-theoretical approaches may enhance explanatory power. A systematic review of theory-based PA interventions in college students identified SCT and TTM as the most frequently applied frameworks and recommended their integration to improve intervention effectiveness (Alshagrawi and Alsayed, 2025). Supporting this approach, Yu et al. (2024) found that self-efficacy mediated associations between PA and psychological traits, with gender-specific patterns, while Fang et al. (2025) reported that a positive association between PA and self-efficacy among Chinese college students.

Accordingly, this study aimed to integrate SCT and TTM to better understand PA determinants, 24-h movement behaviors, and CRF among Chinese college students. Specifically, the study had two aims: (1) to compare PA determinants, 24-h movement behaviors, and CRF across stages of change; and (2) to examine associations between psychosocial determinants of PA (e.g., self-efficacy, decisional balance, processes of change, and social support) and both movement behaviors (Jin et al., 2024) and CRF.

## 2 Theoretical framework: an integrated SCT–TTM perspective

This study is grounded in an integrated theoretical framework combining SCT and TTM to explain PA determinants, 24-h movement behaviors, and CRF among college students. These frameworks were selected because they address complementary dimensions of behavior change: SCT specifies the psychosocial and environmental mechanisms that regulate behavior (Bandura, 1986), whereas TTM characterizes individuals’ readiness for change and progression over time (Prochaska and DiClemente, 1982; Marcus and Forsyth, 2003; Edberg, 2007; Xiong et al., 2017). Integrating these frameworks enable a more comprehensive understanding of both behavioral regulation and behavioral readiness, which is particularly relevant for PA in young adult populations.

SCT posits that behavior is shaped through reciprocal interactions among personal factors, behavior, and the environment (Bandura, 1986). Within PA research, SCT has been widely used to explain initiation and maintenance of active lifestyles, with self-efficacy identified as a central determinant (D’Alonzo et al., 2004; Egele et al., 2025). Self-efficacy reflects individuals’ confidence in their ability to be physically active despite barriers and has demonstrated robust associations with PA participation, self-regulation, and fitness outcomes across diverse populations, including college students (Gao et al., 2008; Hu et al., 2025; Maadleh et al., 2025). Additional SCT constructs, such as enjoyment, social support, and perceived environmental support, facilitate sustained PA engagement by reinforcing positive outcome expectations and enabling self-regulatory strategies (Oyibo et al., 2018). In this study, SCT provides a framework for understanding how PA determinants are associated with 24-h movement behaviors and CRF.

Meanwhile, TTM conceptualizes PA adoption as a dynamic process unfolding across discrete stages of change, ranging from precontemplation to maintenance (Prochaska and DiClemente, 1982). The model emphasizes that individuals at different stages vary in motivational orientation and in their use of decisional balance (perceived pros and cons of PA) and processes of change (cognitive and behavioral strategies supporting progression). Empirical evidence consistently shows that self-efficacy, decisional balance, and PA strategies differ across stages and are particularly influential during transitions from inactivity to regular PA (Kim and Cardinal, 2009; Xie et al., 2025). Therefore, TTM offers a temporal framework for understanding when specific PA determinants are most salient.

In this study, SCT and TTM were integrated by conceptualizing the stage of change as a contextual condition that shapes the expression and influence of SCT-based PA determinants. Specifically, individuals in more advanced stages of change are expected to report stronger self-efficacy, greater use of PA strategies, and more favorable decisional balance, which in turn are associated with healthier 24-h movement behavior profiles and higher CRF. Accordingly, the integrated model proposes that stage-based readiness influences psychosocial determinants (e.g., self-efficacy, PA strategies, and social support), which are in turn associated with movement behaviors and CRF. Within this integrated model, TTM explains readiness and progression, while SCT explains the psychosocial mechanisms through which PA is regulated and maintained.

The theoretical contribution of this study lies in extending the SCT–TTM integration beyond self-reported PA to include objectively measured CRF and movement behaviors across the full 24-h day. By empirically examining how stage-based readiness and SCT constructs jointly relate to 24-h movement behaviors and fitness, this study advances theoretical understanding of how motivational readiness translates into objectively measured behaviors and physiological outcomes. This integrated perspective provides a stronger conceptual basis for the development of stage-tailored, mechanism-focused PA interventions in young adult populations.

## 3 Materials and methods

### 3.1 Procedures

The study was approved by the university’s ethics committee (Approval No.: 20170606P1), and all participants provided written informed consent prior to data collection. Data were collected during regularly scheduled physical education classes in June and July 2017. Participation was voluntary and no academic incentives were provided. During class, participants completed questionnaires assessing demographic characteristics and PA determinants, including stage of change, self-efficacy, decisional balance, processes of change, and social support. Anthropometric measures and CRF testing were conducted during the same session by trained research staff.

### 3.2 Participants

A total of 220 college students (105 males; mean age = 20.15 ± 1.01 years) from a public university in south-central China participated. Data collection occurred in the summer of 2017. Eligibility criteria were: (1) aged between 18 and 25 years; (2) body mass index (BMI) ≥ 18.5 kg/m^2^; (3) no self-reported physical or mental disability that would limit safe participation; and (4) provision of informed consent.

### 3.3 Outcome variables

*Demographic and anthropometric characteristics.* Participants self-reported date of birth and sex. Height (centimeters; cm) was measured to the nearest 0.1 cm using an electronic stadiometer (Seca, Hamburg, Germany). Body mass (kilograms; kg) and body fat percentage were measured using a digital scale (Tanita, Tokyo, Japan). BMI was calculated as weight (kg)/ height (m^2^). BMI categories followed Chinese standards: healthy weight (<24 kg/m^2^) and overweight/obese (≥24 kg/m^2^) (Zhou et al., 2002).

*SCT variables*. Self-efficacy for PA was assessed using a six-item scale reflecting confidence in being active despite common barriers (e.g., fatigue, time constraints). Responses ranged from 1 (not at all confident) to 5 (extremely confident) (Carlson et al., 2012). In the present study, the scale demonstrated good internal consistency (Cronbach’s *α* = 0.79), exceeding the conventional threshold for acceptable reliability (≥0.70). Enjoyment of PA was assessed using a four-item measure rated on a 5-point Likert scale from 1 (strongly disagree) to 5 (strongly agree). The scale demonstrated good internal consistency in this sample (Cronbach’s *α* = 0.81). In this study, social support included family support and friend support. Family support was measured using a four-item scale and friend support was assessed with a five-item scale using similar items. Both were rated on a frequency scale from 1 (almost never) to 5 (every day). Internal consistency was acceptable for both family support (Cronbach’s *α* = 0.78) and friend support (Cronbach’s *α* = 0.80). Perceived physical environment was assessed using a four-item scale rated from 1 (strongly disagree) to 5 (strongly agree) (Carlson et al., 2012). The scale demonstrated acceptable internal consistency (Cronbach’s *α* = 0.74).

*TTM variables*. Stage of change for physical activity was assessed using a validated stage classification algorithm based on TTM (Marcus and Forsyth, 2003). Participants were categorized into one of five stages (precontemplation, contemplation, preparation, action, or maintenance) based on their current PA behavior and intention to be regularly physically active (≥150 min of moderate-to-vigorous PA per week). For analytic purposes and consistent with prior research in college populations, participants were further grouped into low stage (precontemplation, contemplation, preparation) and high stage (action, maintenance) categories (Xiong et al., 2017; Oba et al., 2024). Decisional balance was assessed using a 10-item measure capturing perceived advantages (pros; e.g., “I would have more energy…”) and disadvantages (cons; e.g., “…would take too much time”) of engaging in PA, with responses ranging from 1 (not important) to 5 (extremely important). The overall scale demonstrated acceptable internal consistency in the present sample (Cronbach’s *α* = 0.76). Processes of change (PA strategies) were assessed using a 15-item measure assessing behavioral and cognitive strategies (e.g., “I look for information about…” or “I say positive things to myself about…”), rating from 1 (never) to 5 (many times) (Saelens et al., 2000; Carlson et al., 2012). Internal consistency for this scale was good (Cronbach’s *α* = 0.83).

*Movement behaviors.* PA and Sedentary Time (ST) were assessed using ActiGraph Link GT9X accelerometers (ActiGraph, Pensacola, FL) (McClain et al., 2007). Raw acceleration data were sampled at 30 Hz and processed using ActiLife software (Version 6.13.3). Non-wear time was identified using the ActiGraph default algorithm, defined as ≥90 consecutive minutes of zero activity counts, allowing for up to 2 min of non-zero counts. Valid wear days required ≥12 h of wear time, with inclusion criteria of at least two valid weekdays and one valid weekend day, consistent with established protocols (Christiansen et al., 2023; Freedson et al., 1997; Giurgiu et al., 2024; Kelly et al., 2013). Activity intensity was classified using validated wrist-worn cut-points for adults, generating daily estimates of sedentary behavior, light physical activity (LPA), and moderate-to-vigorous physical activity (MVPA) (Clevenger et al., 2024). Daily averages across valid days were used in all analyses. Daily averages for LPA, MVPA, and ST were calculated and used as study outcomes.

*Sleep efficiency and sleep duration*: Sleep duration and efficiency were derived from wrist-worn accelerometer data using the Sadeh sleep–wake algorithm implemented in ActiLife software, which has demonstrated validity in young adult populations (Sadeh et al., 1994). Sleep periods were identified using a combination of accelerometer data and participant-reported sleep diaries to define bedtime and wake time. Sleep duration was calculated as total sleep time per night, and sleep efficiency was defined as the ratio of total sleep time to time in bed. Poor sleep was operationalized as <7 h of sleep duration or sleep efficiency <85%, consistent with prior research.

*Cardiovascular fitness*. CRF was assessed using the YMCA 3-Minute Step Test. Following 5 minutes of seated rest, baseline heart rate was recorded via radial pulse palpation. Participants then completed 3 minutes of stepping at a cadence of 96 beats per minute on a 12-inch bench. Immediately upon completion, participants sat down and 1-min recovery heart rate was measured. Recovery heart rate served as the primary CRF indicator, with lower recovery heart rate values indicating higher cardiovascular fitness, consistent with established protocols (Levy and Cardinal, 2006).

*Psychosocial measures and validation.* All psychosocial scales used in this study were derived from established instruments with demonstrated reliability and validity in physical activity research (Saelens et al., 2000; Carlson et al., 2012). These measures have been previously used or culturally adapted in Chinese or East Asian college student samples, demonstrating acceptable internal consistency and construct validity (Xiong et al., 2017; Fang et al., 2025). In the present study, all scales exhibited acceptable internal consistency (Cronbach’s *α* ≥ 0.70), supporting their use in this population.

Data were collected in 2017 prior to the widespread adoption of consumer wearables and digital health platforms; however, objective accelerometer-based assessment and theory-driven psychosocial measures remain consistent with current best practices in PA research.

### 3.4 Data analyses

All tables and statistical outputs were re-checked to ensure internal consistency of sample sizes, test statistics, *p*-values, and significance indicators. Data analyses were conducted in four steps via SPSS (version 29.0). To address Aim 1, participants were grouped into low stage (precontemplation, contemplation, preparation) and high stage (action, maintenance) categories. This grouping strategy was adopted to ensure adequate statistical power within groups and is consistent with prior PA research applying the TTM in college populations (Marcus and Forsyth, 2003; Xiong et al., 2017; Oba et al., 2024). Given the modest sample size within certain individual stages, collapsing stages was deemed appropriate to reduce instability in parameter estimates while preserving meaningful distinctions between inactive and active behavioral profiles. One-way analysis of variance (ANOVA) and multivariate analyses of variance (MANOVA) were used to examine stage-group differences in CRF, PA determinants, and 24-h movement behaviors. Prior to multivariate analyses, assumptions of normality and homogeneity of variance–covariance matrices were evaluated using skewness indices and Box’s *M* tests.

To address Aim 2, Pearson correlations and simultaneous multiple regression analyses were conducted to examine associations among PA determinants, movement behaviors, and CRF. Predictors entered the regression models included self-efficacy, decisional balance, PA strategies, enjoyment, social support, perceived environment, and relevant movement behaviors, depending on the outcome variable. Multicollinearity was assessed using variance inflation factors (VIF), with all values below 5.0, indicating no concern for multicollinearity. Regression assumptions—including linearity, normality of residuals, homoscedasticity, and independence—were evaluated and met. Given the co-dependent nature of 24-h movement behaviors, results from regression analyses should be interpreted as associational rather than predictive. Statistical significance was set at *p* < 0.05.

## 4 Results

### 4.1 Descriptive analyses

Descriptive statistics for all study variables are presented in Table 1. Internal consistency for all PA determinant measures was acceptable, with Cronbach’s alpha coefficients exceeding 0.70. Table 1 summarizes participant characteristics, PA determinants, 24-h movement behaviors, sleep outcomes, and CRF. Overall, participants reported relatively high levels of PA strategies, decisional balance, self-efficacy, PA enjoyment, and perceived physical environment, as mean scores of these variables exceeded the scale midpoint (2.50). Yet, they reported modest level of social support (i.e., family support and friend support).

**Table 1:** Descriptive statistics of the study variables.

Variable (unit)	N	Mean	Standard deviation
Self-efficacy	220	2.55	0.79
PA enjoyment	220	3.48	0.97
Family support	219	2.04	0.78
Friend support	220	2.23	0.68
Physical environment	220	3.01	0.65
PA strategies	219	2.97	0.57
Decision balance	219	3.56	0.42
Sedentary behavior (minute)	201	689.96	150.32
Light PA (minute)	203	105.85	29.77
MVPA (minute)	203	93.93	28.83
Sleep duration (minute)	215	340.44	78.77
Sleep efficiency (%)	216	84.12	4.79
CRF	220	106.08	16.54
Age (years old)	220	20.16	1.01
Height (cm)	218	164.78	10.00
Weight (kg)	217	57.84	13.25
BMI	220	20.67	3.12
Body Fat Percentage (%)	220	21.92	7.94

### 4.2 Group differences across stage of change

Descriptive statistics for PA determinants, 24-h movement behaviors, and CRF across low- and high-stage groups are presented in Table 2. An ANOVA indicated no significant difference in CRF between stage groups, *F*(1, 218) = 1.29, *p* = 0.25, *η*^2^ = 0.006.

**Table 2:** Descriptive statistics among the outcome variables across groups (*N* = 217).

Variable	Low stage			High stage		
	Mean	SD	*N*	Mean	SD	*N*
Self-efficacy	2.38	0.71	158	2.99	0.83	59
PA enjoyment	3.33	0.88	158	3.88	1.07	59
Family support	2	0.74	158	2.11	0.89	59
Friend support	2.14	0.64	158	2.51	0.75	59
Physical environment	2.96	0.64	158	3.11	0.69	59
PA strategies	2.89	0.55	158	3.18	0.57	59
Decision balance	3.51	0.41	158	3.68	0.41	59
Sedentary behavior	696.99	133.54	145	671.75	187.11	56
Light PA	106.27	28.3	145	104.50	33.96	56
MVPA	93.95	28.15	145	93.42	31.15	56
Sleep duration	338.96	68.13	145	336.36	84.25	56
Sleep efficiency	83.71	4.09	145	84.27	5.38	56
CRF	106.84	15.67	161	103.99	18.70	59

SD = standard deviation.

For the assumptions of multivariate normality and homogeneity of variance–covariance matrices, the values of skewness ranged from −0.58 to −1.26, suggesting the outcome variables were approximately normally distributed. In addition, the Box *M* test yielded no violation of the assumption of homogeneity of variance–covariance matrices (*p* > 0.05). A MANOVA examining stage-group differences in 24-h movement behaviors revealed no significant multivariate effect, Wilks’ *Λ* = 0.99, *F*(5,195) = 0.39, *p >* 0.05, *η*^2^ = 0.10. In contrast, a MANOVA examining PA determinants revealed a significant multivariate effect for stage group, Wilks’ *Λ* = 0.84, *F*(7,209) = 5.53, *p <* 0.001, *η*^2^ = 0.16. Follow-up ANOVA analyses (see Table 2) indicated significant stage group differences in self-efficacy, decisional balance, PA strategies, friend support, and enjoyment (*p* < 0.01).

### 4.3 Correlation and regression analyses

Bivariate correlations among PA determinants, 24-h movement behaviors, sleep outcomes, and CRF are presented in Table 3. Self-efficacy demonstrated low to moderately positive correlations with other PA determinants (*r* = 0.17–0.62, *p* < 0.05) and CRF and negatively correlated with ST. Most PA determinants were moderately positively correlated with one another (*r* = 0.14–0.53, *p* < 0.05). Enjoyment and decisional balance were significantly positively correlated with MVPA, while decisional balance was also positively correlated with LPA. Movement behaviors were significantly and positively related to one another (*r* = 0.55–0.87*, p* < 0.05), whereas CRF was not significantly correlated with movement behaviors.

**Table 3:** Correlation analyses among the outcome variables (*N* = 220).

Variables	1	2	3	4	5	6	7	8	9	10	11	12	13
1. Self-efficacy	–												
2. PA enjoyment	0.49*	–											
3. Family support	0.29*	0.04	–										
4. Friend support	0.35*	0.29*	0.53*	–									
5. Physical environment	0.17*	0.14*	0.11	0.16*	–								
6. PA strategies	0.62*	0.44*	0.40*	0.36*	0.20*	–							
7. Decision balance	0.26*	0.40*	−0.05	0.10	0.08	0.35*	–						
8. Sedentary	−0.15*	0.004	−0.07	−0.09	0.001	−0.03	0.11	–					
9. Light PA	−0.04	0.13	−0.03	0.01	0.03	0.04	0.22*	0.59*	–				
10. MVPA	0.005	0.16*	−0.02	−0.02	0.07	0.06	0.26*	0.55*	0.87*	–			
11. Sleep duration	−0.01	−0.02	−0.002	0.07	0.04	−0.06	−0.08	−0.35*	−0.46*	−0.47*	–		
12. Sleep efficiency	−0.001	0.07	−0.11	0.02	0.07	−0.08	0.10	−0.45*	−0.28*	−0.32*	0.31*	–	
13. CRF	0.15*	0.12	−0.04	−0.01	0.07	0.08	0.11	−0.01	0.05	0.14*	−0.13	0.07	–

*p < 0.05.

To examine the predictive utility of students’ PA determinants and/or other relevant outcomes to the three dependent variables (CRF, MVPA, and sleep efficiency), we conducted three simultaneous multiple regression analyses. In the CRF model, MVPA (*t* = 1.96, *p* = 0.05) demonstrated a significant association with CRF, while self-efficacy showed a marginal association (*t* = 1.84, *p* = 0.06). In the MVPA model, LPA was significantly associated with MVPA (*t* = 16.73, *p* < 0.01), whereas sleep efficiency was negatively associated with MVPA (*t* = −2.00, *p* < 0.01). In the sleep efficiency model, both MVPA (*t* = −2.00, *p* < 0.05) and ST (*t* = −4.87, *p* < 0.01) were significantly associated with sleep efficiency (See Table 4).

**Table 4:** Results of regression analyses on college students’ outcomes.

Variables in equation	*R* ^2^	*β*	*p*	*t*
CRF				
Model	0.10			
Self-efficacy		0.18	0.06*	1.84
MVPA		0.29	<0.05	1.96
MVPA				
Model	0.78			
Light PA		0.80	<0.01	16.73
Sleep efficiency		−0.08	<0.05	−2.00
Sleep efficiency				
Model	0.27			
MVPA		−0.26	<0.05	−2.00
Sedentary behavior		−0.36	<0.01	−4.87

*β* values are standardized regression coefficients from the final stage of the regression analysis. *: marginal significant effect.

## 5 Discussion

This study applied an integrated framework combining SCT and TTM to examine PA determinants, 24-h movement behaviors, and CRF among Chinese college students. The primary finding was a clear divergence between psychosocial and behavioral outcomes: students in more advanced stages of change reported stronger PA determinants—particularly self-efficacy, decisional balance, PA strategies, friend support, and enjoyment—yet objectively measured movement behaviors and CRF did not differ by stage. At the same time, multivariable analyses indicated that MVPA and self-efficacy were most strongly associated with CRF, and that movement behaviors exhibited interdependent relationships within a 24-h framework. By incorporating objective movement behavior assessment and fitness testing, the study extends prior theory-based research that has largely relied on self-reported PA outcomes. Overall, findings reveal a meaningful divergence between psychosocial readiness for PA and objectively measured behavioral and physiological outcomes, offering important insights into how motivational constructs translate into health-relevant behaviors in young adults (Kracht et al., 2024; Oba et al., 2024; Alshagrawi and Alsayed, 2025).

### 5.1 Stage differences in PA determinants, movement behaviors, and fitness

Consistent with TTM, students in higher stages of change reported significantly stronger PA determinants, including self-efficacy, decisional balance, PA strategies, enjoyment, and friend support. These findings align with prior stage-based research demonstrating that individuals in action and maintenance stages exhibit more favorable motivational profiles and greater use of self-regulatory strategies than those in earlier stages (Marcus and Forsyth, 2003; Xiong et al., 2017; Oba et al., 2024). Consistent with theory, progression through stages of change may first emerge in psychosocial readiness and self-regulatory processes before being reflected in objectively measured behavioral differences. From an SCT perspective, higher stages of change may reflect accumulated mastery experiences and positive outcome expectations, which reinforce confidence and engagement in PA-related behaviors.

However, contrary to theoretical expectations, no stage-based differences were observed in accelerometer-derived movement behaviors or CRF. This pattern warrants careful interpretation. First, it highlights a critical distinction between psychological readiness and actual behavioral execution, particularly when PA is assessed objectively. While TTM assumes that progression through stages corresponds with increased behavior enactment, emerging evidence suggests that this relationship is weaker when PA is measured using devices rather than self-report (Christiansen et al., 2023; Clevenger et al., 2024). Wearable monitoring may reduce social desirability bias and compress variability across groups, thereby attenuating stage-based differences.

Second, contextual factors specific to college environments may partially explain these findings. Shared academic schedules, campus walkability, and structured physical education requirements may impose similar movement demands across students regardless of motivational stage, resulting in relatively homogeneous activity patterns. Prior research suggests that environmental constraints can override individual-level motivation, particularly in young adult populations with limited autonomy over daily routines (Brown et al., 2024). Finally, ceiling effects may have contributed to null stage differences. Participants demonstrated relatively high MVPA levels and favorable fitness profiles, which may limit the capacity to detect between-group differences in behavior or CRF. Similar patterns have been reported in studies applying 24-h movement frameworks to healthy young adult samples (Kracht et al., 2024).

Taken together, these findings suggest that stage of change more strongly differentiates motivational and regulatory constructs than objectively measured behaviors in this population. This underscores the importance of integrating SCT constructs—such as self-efficacy and self-regulation—into stage-tailored interventions to help translate readiness into sustained behavioral change.

### 5.2 Associations among PA determinants, movement behaviors, and CRF

Regression analyses revealed that MVPA was the strongest behavioral predictor of CRF, while self-efficacy showed a marginal association with fitness outcomes. These findings align with SCT’s central proposition that confidence in one’s capabilities supports sustained engagement in health-enhancing behaviors, which in turn drive physiological adaptation (Bandura, 1986). Notably, the CRF regression model explained a relatively small proportion of variance, underscoring that CRF is a multifactorial outcome and that psychosocial and behavioral variables alone provide only a partial account of fitness differences in this sample. Similar associations between self-efficacy, MVPA, and fitness have been reported in college and young adult populations (Li et al., 2022; Yu et al., 2024).

Notably, PA determinants were weakly or inconsistently associated with accelerometer-derived movement behaviors. This discrepancy mirrors prior research showing that psychosocial constructs often demonstrate stronger associations with self-reported PA than with device-measured activity (Christiansen et al., 2023; Fiedler et al., 2025). Measurement sensitivity, cut-point selection, and limited contextual specificity of psychosocial instruments may contribute to this attenuation. Additionally, psychosocial measures may better capture intentional exercise behavior, whereas accelerometers record total movement accumulated across diverse contexts, including non-volitional activity.

The strong positive association between LPA and MVPA suggests that lower-intensity movement may serve as a behavioral gateway to more vigorous activity, consistent with both SCT’s emphasis on mastery experiences and emerging 24-h movement behavior frameworks. Incremental increases in movement may reinforce perceived competence, thereby facilitating engagement in higher-intensity PA over time (Li et al., 2025).

The observed inverse associations between MVPA, ST, and sleep efficiency further highlight the interdependent nature of 24-h movement behaviors. While PA is generally associated with improved sleep quality, excessive intensity, late-day exercise, or time-use trade-offs may temporarily compromise sleep efficiency among college students (Yin et al., 2024; Zhang et al., 2025). The findings emphasize that PA promotion efforts should consider behavioral balance across the full day, rather than focusing exclusively on MVPA.

### 5.3 Implications for theory, practice, and future research

Theoretically, this study advances SCT–TTM integration by demonstrating that motivational readiness and psychosocial regulation are more strongly aligned with each other than with objectively measured behavior. This finding suggests that readiness alone may be insufficient to predict movement behavior without targeted support for self-regulation and environmental facilitation.

From a practical standpoint, PA interventions for college students may benefit from combining stage-matched strategies (TTM) with mechanism-focused components (SCT), such as enhancing self-efficacy through mastery experiences, leveraging peer support, and promoting incremental increases in daily movement. Importantly, interventions should also address sedentary behavior and sleep concurrently within a 24-h framework.

Future research should employ longitudinal and experimental designs to test causal pathways linking stage of change, SCT constructs, movement behavior composition, and CRF (Waki et al., 2024). Greater emphasis on culturally sensitive measurement tools and harmonized accelerometer protocols may further clarify psychosocial–behavioral linkages in diverse college populations.

### 5.4 Strengths and limitations

Key strengths of this study include a sizeable sample of Chinese college students—a historically understudied population—and objective assessments of PA, ST, sleep, and CRF. However, the cross-sectional design precludes causal inference regarding the directionality of associations among psychosocial determinants, movement behaviors, and fitness. Future studies should employ randomized trials and longitudinal designs that (a) stage-match TTM-based strategies, (b) explicitly target self-efficacy and self-regulation through SCT-informed approaches, and (c) analyze time-use composition to capture interdependent trade-offs among MVPA, LPA, ST, and sleep (Alshagrawi and Alsayed, 2025; Kracht et al., 2024; Xie et al., 2025). Methodological efforts to harmonize accelerometer processing (e.g., wrist cut-points, epoch lengths) and addressing selection/reactivity biases will improve comparability and sensitivity to change in university settings (Christiansen et al., 2023; Clevenger et al., 2024).

Several methodological considerations should be noted. First, although the TTM conceptualizes behavior change across five stages, stages were collapsed into two groups for analysis. This approach, while common in PA research, may have reduced sensitivity to detect more nuanced stage-specific differences. However, collapsing stages were necessary to maintain statistical power and reduce estimation instability given sample size constraints. Future studies with larger samples should examine stage-specific patterns across all five stages. Second, 24-h movement behaviors are inherently co-dependent, such that increases in one behavior necessitate decreases in others.

Several descriptive patterns in the movement behavior data warrant clarification. First, the relatively high levels of MVPA observed in this sample may reflect the use of wrist-worn accelerometers, which tend to yield higher MVPA estimates compared to hip-worn devices, particularly when applied in free-living young adult populations. Wrist placement is more sensitive to upper-limb movement and non-exercise activities, which can inflate MVPA estimates relative to self-report or hip-based protocols. In addition, the sample consisted of college students enrolled in physical education courses, which may further contribute to elevated activity levels. Also, average sleep duration appeared lower than recommended guidelines. This finding is consistent with prior evidence indicating that college students frequently experience short sleep duration due to academic demands, irregular schedules, and technology use (Dunietz and Jansen, 2026). Wrist-based accelerometry may also underestimate sleep duration (de Zambotti et al., 2019) when periods of quiet wakefulness are misclassified as non-sleep, particularly in young adults with fragmented sleep patterns.

While standard regression approaches provide useful preliminary insights, they do not fully capture this compositional structure. For example, positive correlations between sedentary behavior and both LPA and MVPA likely reflect the co-dependent nature of time-use data, rather than contradictory behavioral patterns. Individuals who accumulate more total wear time or engage in longer waking hours may simultaneously record higher volumes of sedentary time and physical activity. Such correlations are well-documented in accelerometer research and highlight limitations of traditional correlational and regression approaches when applied to 24-h movement behaviors. Consequently, findings related to movement behaviors should be interpreted with caution. Future research employing compositional data analysis or isotemporal substitution models may provide more precise estimates of behavior trade-offs.

Also, the cross-sectional design precludes causal inference. Several psychosocial and behavioral variables were associated with movement behaviors and cardiovascular fitness, effect sizes were modest and models explained limited variance, particularly for CRF. Associations observed in this study should not be interpreted as directional or predictive, but rather as reflecting concurrent relationships among PA determinants, movement behaviors, and CRF. Several sampling limitations should be considered. Participants were drawn from a single public university in south-central China, which may limit generalizability to other regions, institutions, or cultural contexts where activity opportunities and academic demands differ. Underweight students were excluded to ensure safety and reduce confounding, but this may limit applicability to individuals with lower BMI who may have different activity patterns. Participation was voluntary, introducing potential self-selection bias, as more health-conscious students may have enrolled, possibly reducing variability in outcomes. Future studies should use multi-site, diverse samples and targeted recruitment to improve generalizability and better represent broader student populations.

Finally, a limitation is that data were collected in 2017. However, the findings remain relevant because the core psychosocial constructs are stable PA predictors grounded in established theories (SCT, TTM) and have shown consistency over time. While wearable technologies have advanced, fundamental movement behaviors and accelerometer-based assessments remain standard and comparable to current practices. Although modern contexts (e.g., increased smartphone use) may influence PA levels, they are unlikely to alter underlying theoretical relationships. Thus, findings inform mechanisms rather than current prevalence, and future studies should use updated cohorts to confirm these relationships.

## 6 Conclusion

This study provides evidence that Chinese college students in more advanced stages of PA behavior change report stronger PA determinants, including self-efficacy, decisional balance, and self-regulatory strategies, even when objectively measured movement behaviors and CRF do not differ by stage. Associations among MVPA, selected psychosocial constructs, and CRF were modest, suggesting that these factors represent partial correlates rather than primary drivers of fitness in this population. Collectively, the findings support the relevance of integrating stage-based readiness with social-cognitive mechanisms when examining PA behavior, while underscoring the need for longitudinal and experimental research to clarify causal pathways and strengthen explanatory models.

## Data Availability

The raw data supporting the conclusions of this article will be made available by the authors, without undue reservation.
